# Characterization of a New CAMP Factor Carried by an Integrative and Conjugative Element in *Streptococcus agalactiae* and Spreading in Streptococci

**DOI:** 10.1371/journal.pone.0048918

**Published:** 2012-11-09

**Authors:** Sarah Chuzeville, Aurore Puymège, Jean-Yves Madec, Marisa Haenni, Sophie Payot

**Affiliations:** 1 Unité Antibiorésistance et Virulence Bactériennes, Anses Lyon, Lyon, France; 2 INRA, UMR1128 Génétique et Microbiologie, Faculté des Sciences et Technologies, Bd des Aiguillettes BP70239, Vandœuvre-lès-Nancy, France; 3 Université de Lorraine, UMR1128 Génétique et Microbiologie, Faculté des Sciences et Technologies, Bd des Aiguillettes BP70239, Vandœuvre-lès-Nancy, France; University of Kansas Medical Center, United States of America

## Abstract

Genetic exchanges between Streptococci occur frequently and contribute to their genome diversification. Most of sequenced streptococcal genomes carry multiple mobile genetic elements including Integrative and Conjugative Elements (ICEs) that play a major role in these horizontal gene transfers. In addition to genes involved in their mobility and regulation, ICEs also carry genes that can confer selective advantages to bacteria. Numerous elements have been described in *S. agalactiae* especially those integrated at the 3′ end of a tRNA^Lys^ encoding gene. In strain 515 of *S. agalactiae*, an invasive neonate human pathogen, the ICE (called *515_tRNA^Lys^*) is functional and carries different putative virulence genes including one encoding a putative new CAMP factor in addition to the one previously described. This work demonstrated the functionality of this CAMP factor (CAMP factor II) in *Lactococcus lactis* but also in pathogenic strains of veterinary origin. The search for co-hemolytic factors in a collection of field strains revealed their presence in *S. uberis*, *S. dysgalactiae*, but also for the first time in *S. equisimilis* and *S. bovis*. Sequencing of these genes revealed the prevalence of a species-specific factor in *S. uberis* strains (Uberis factor) and the presence of a CAMP factor II encoding gene in *S. bovis* and *S. equisimilis*. Furthermore, most of the CAMP factor II positive strains also carried an element integrated in the tRNA^Lys^ gene. This work thus describes a CAMP factor that is carried by a mobile genetic element and has spread to different streptococcal species.

## Introduction

*S. agalactiae* (group B Streptococci or GBS) is a human and animal opportunistic invasive pathogen. Cow mastitis caused by *S. agalactiae* was reported in 1887, while human infections were observed only 50 years later. GBS causes mastitis in cattle and septicemia, meningitis and endocarditis in humans [1]–[4]. It produces various virulence factors implicated in the adhesion and colonization steps, immune evasion and adaptation to the host environment [5], [6]. In particular, GBS develops a characteristic arrow-shaped hemolysis when cultivated near *Staphylococcus aureus*, which is said to be due to an exosubstance named CAMP factor [7], [8]. In fact, CAMP factor causes cell lysis when erythrocytes have been first sensitized by incubation with sphingomyelinase (SMase), a protein secreted by *S. aureus* and numerous other bacterial species [9]. SMases hydrolyse sphingomyelin, an erythrocyte membrane component, thus weakening the membrane if it contains at least 45% of sphingomyelin [10]. Therefore, rabbit, mouse and human erythrocytes (19, 25 and 27% of sphingomyelin) are not sensitive to CAMP factor, while goat, sheep and dairy cow erythrocytes (46, 51 and 52%, respectively) are [10], [11]. The CAMP factor does not have an enzymatic activity but experiments showed that monomers could bind and oligomerize membrane components, in particular glycosylphosphotidylinositol (GPI) anchored proteins, thus forming a pore in the erythrocyte membrane [7], [12].

Classification of the CAMP factor as a virulence factor according to the Koch postulates remains controversial since some authors have shown that injection of purified CAMP factor could increase mortality of rabbits and mice [13], [14], while other authors could not demonstrate any effect of a deletion of the CAMP factor encoding gene (*cfb*) on GBS pathogenicity [15]. The *cfb* gene is ubiquitous in GBS strains so that CAMP test or search of the *cfb* gene by Polymerase Chain Reaction (PCR) was usually used to differentiate GBS from other *Streptococcus* species [16]–[19]. However, CAMP factor homologues were described in other Gram positive species such as *S. pyogenes* [20], *S. uberis* [21], *S. porcinus* [22], *S. canis* [23] and *Propionibacterium acnes* [24].

In bacteria, horizontal transfers promote rapid genome evolution and may be involved in speciation [25]–[27]. In this way, 18% of the *Escherichia coli* genome has been acquired by horizontal transfer during the past 100 million years [28]. Mobile genetic elements include conjugative plasmids, phages, transposons, and Integrative and Conjugative Elements (ICEs). ICEs represent a family of mobile genetic elements consisting of clustered genes able to direct their own excision, transfer by conjugation and integration into the chromosome of recipient cell [29], [30]. The integration of the element is most often site-specific. Recombination between repeated sequence sites *attR* and *attL* (R for right and L for Left) bordering the ICE brings into play an integrase by a mechanism similar to bacteriophage integration. The transfer displays similarity with conjugative plasmid transfer. In the recipient cell, the integrase catalyzes recombination between the *attI* site present on the circular shape of the ICE and the *attB* site present at target chromosomal site. After transfer, both donor and recipient cells have a copy of the ICE in their genome [29], [30].

Interestingly, most of the ICEs already described encode additional functions conferring advantageous properties to the host strain, such as antimicrobial resistance, virulence or environmental adaptation [31]. Numerous works have shown that ICEs contribute to genome flexibility and that mobile genetic elements are prevalent in Streptococcus species such as *S. agalactiae* [32], [33], *S. pyogenes* [34] and *S. pneumoniae* [35]. Genome analyses showed that *S. uberis* and *S. suis* genomes carry fewer mobile genetic elements than other streptococci, suggesting that it could restrict the acquisition of exogenous genetic elements in these species [36], [37].

Sequencing of 8 GBS strain genomes, belonging to different serotypes causing major infections, highlighted the diversity of its variable gene pool [32]. Thirty five ICEs or related elements were detected, one hotspot of integration being the 3′ extremity of a tRNA^Lys^ gene [33]. Many of the proteins encoded by these elements share a high identity with proteins produced by *S. pyogenes*, *S. pneumoniae* or *S. dysgalactiae* subsp. *equisimilis*. ICE prevalence in *S. agalactiae* was confirmed in various field strains belonging to different clonal complexes in particular for the ICE integrated at the tRNA^Lys^ site which was also detected in other streptococci [33], [38].

A GBS strain isolated from cattle infection was sequenced and genome analysis revealed distinct genetic diversity when compared with strains from humans [39]. Data suggest that genetic exchanges between veterinary and human strains may be infrequent, except in case of prolonged contact [32], [40]. Demonstration of transfers between this bovine strain of *S. agalactiae* and other cattle pathogens (*S. uberis* and *S. dysgalactiae*) suggests a high rate of horizontal transfer between strains occurring in the same environment [39]. Thus, interspecies transfer is likely to be frequent, thus enabling the transfer of functions useful for the adaptation to the host environment [41]–[45].

This work aims to characterize a putative new CAMP factor carried by an ICE (ICE_*515_tRNA^Lys^*) of *S. agalactiae*. ICE_*515*_*tRNA^Lys^* was recently demonstrated to be functional and able to autonomously transfer by conjugation to other S. *agalactiae* strains (A. Puymege, submitted for publication).

Here, we demonstrated the functionality of this CAMP factor II in non pathogenic and pathogenic strains. Our work also highlights the spread of CAMP factor II among other field pathogenic streptococci of veterinary origin, as expected for a mobile genetic element.

## Materials and Methods

### Bacterial strains, media and growth conditions

The 515 GBS strain of serotype Ia [32] and NEM316 GBS strain of serotype III [46], two invasive neonate human pathogenic strains were used in this study (GenBank accession no. PRJNA54311 and PRJNA334 respectively). Strain 515 was isolated from the cerebrospinal fluid of an infected patient while strain NEM316 was isolated from a case of fatal septicaemia. The field pathogenic streptococci used (n = 677) were isolated through the National Network for the Surveillance of Resistance to Antimicrobials in Animals in France (Resapath, www.resapath.anses.fr) between 1984 and 2011. This collection includes isolates of *S. uberis* (n = 449); *S. dysgalactiae* (n = 162); *S. bovis* (n = 31); *S. suis* (n = 26) and *S. equisimilis* (n = 9). *E. coli* strains were used for the cloning experiments. Strains and their characteristics are listed in Table S1. Streptococci and *E. coli* strains were grown in Brain Hearth Infusion (BHI) (AES CHEMUNEX, Bruz, France) or on Tryptic soy agar plates supplemented with 5% of defibrinated sheep blood (Biomérieux, Marcy l'Etoile, France). Lactococcal strains were grown in M17 medium (Sigma Aldrich, Steinheim, Germany) supplemented with 0.25% glucose either in liquid medium or on agar plates. Antibiotics were used at the following concentrations: kanamycin, 50 µg.mL^−1^ for *E. coli*; erythromycin, 20 µg.mL^−1^ for streptococci and lactococci and 150 µg.mL^−1^ for *E. coli*. Streptococci and lactococci were grown without shaking in flasks at 37°C and 30°C, respectively, while *E. coli* was grown in flasks with shaking at 37°C.

### Transconjugant construction

ICE_*515_tRNA^Lys^* was tagged at its left hand side (in the SAG2026 gene encoding the ATPase part of an ABC transporter) by a resistance gene using the pG+host9spc vector. Vector pG+host9spc is a derivative of the pG+host9 plasmid carrying a spectinomycin resistance gene from the pSET4S plasmid (Bellanger pers. comm.) [47]. To construct the mutant, the 5′ and 3′ ends of SAG2026 gene were independently amplified by PCR (using primers SAG2026-1-*Hin*dIII, SAG2026-2-*Avr*II, SAG2026-3-*Avr*II and SAG2026-4-*Eco*RI). An erythromycin resistance gene amplified from pG+host9 was inserted in the internal *Avr*II site and the whole insert (containing the two SAG2026 fragments separated by the *ery* cassette) was then cloned into pG+host9spc to give pG+host9spc-SAG2026ery, which was used to transform *S. agalactiae* by electroporation [48]. Two crossovers, upstream and downstream of the tagged region, were selected as described previously to obtain replacement of the gene by the *ery* resistance gene [41]. The tagged ICE was then transferred to the recipient strain by filter mating as described by Bellanger *et al.* [41] with minor modifications. Briefly, both donor and recipient strains were grown overnight. A 15 mL-culture of the relevant broth was inoculated with 150 µL of overnight culture of the recipient or the donor strain. Cultures were grown at the relevant temperature until mid-exponential phase (optical density at 600 nm of 0.4). Cultures of the donor and recipient were mixed and centrifuged for 15 min in a prewarmed centrifuge at 4,500× *g*. The pellet was resuspended in 1 mL of BHI broth, and 150 µL aliquots were spread onto 0.45 µm-pore-size nitrocellulose filters (Sartorius, Goettingen, Germany) on tryptic soy blood agar plates which were incubated for 14 h at 37°C. The filters were removed from the agar plates, placed in 50 mL bottles containing 10 mL of sterile BHI broth, and vortexed for 30 s. Various dilutions were spread on blood agar plates supplemented with the appropriate antibiotics, and plates were incubated for 24 h in order to count CFU of the donor, the recipient, and the transconjugants.

### RT-PCR

The expression of the SAL_2074 gene, which encodes the putative new CAMP factor on the ICE_*515_tRNA^Lys^* genetic element, was examined by RT-PCR. Briefly, a 10 mL-preculture was grown overnight at 37°C and seeded at 1∶100 ratio in 10 mL of BHI or M17 liquid medium. Two mL of cell pellets were collected at OD_600_ = 0.4 and 0.8 by centrifugation at 2000× *g* at 4°C and conserved by a quick freezing at −80°C. RNA samples were purified using the RNAeasy mini kit (Qiagen, Hilden, Germany) before being treated with the RQ1 RNase free DNase (Promega, Madison, USA) for 1 hour. DNA was synthesized using the M-MLV retro-transcriptase (In Vitrogen Life Technologies, Carlsbad, USA). Conventional PCR were done using cDNA as templates for DNA polymerase (Roche Applied Science, Mannheim, Germany) as described earlier [49].

### Analysis of the supernatants by SDS-PAGE and mass spectrometry

Supernatant of *S. agalactiae* NEM316 and NEM316 (ICE_*515_tRNA^Lys^*) cultures grown overnight in BHI or chemically defined medium (CDM, peptide-free medium, [50]) were collected after centrifugation at 5000× *g* at 4°C for 10 min. Proteins were precipitated using a sodium deoxycholate (DOC) - trichloroacetic acid (TCA) protocol [51]. Briefly, DOC was added to a final concentration of 0.02% to 30 mL of supernatant. After mixing and a 30 min-sitting on ice, TCA was added to a final concentration of 10% and the proteins were precipitated overnight at 4°C. The mixed protein-detergent precipitate was collected by centrifugation (14,000× *g*, 15 min, 4°C). The supernatant was carefully removed and washed two times with cold acetone (stored at −20°C). Pellets were dried under vacuum and resuspended in SDS gel loading buffer (50 mM Tris HCl pH 6.8, 2% SDS, 10% glycerol, 0.1% bromophenol blue and 100 mM DTT) (to reach a 250-fold concentration for cultures grown in BHI and a 1000-fold concentration for CDM cultures). After a 10 min-denaturation step at 98°C, proteins (15 µL of sample) were size-separated by sodium dodecyl sulphate-polyacrylamide gel electrophoresis (SDS-PAGE) using a 12% acrylamide resolving gel. Bands of interest were excised from the gel and processed as follows for protein content identification. After cysteine reduction (DTT 30 mM, 100 mM ammonium bicarbonate BA) and alkylation (30 mM iodoacetamide in BA), bands were washed twice in BA and BA/acetonitrile (1∶1) and then dried under vacuum before overnight trypsin digestion (in BA). Peptides were then extracted twice in acetonitrile 80%, trifluoroacetic acid (TFA), dried under vacuum and resuspended in a solution of 2% acetonitrile and 0.1% TFA.

HPLC was performed using an Ultimate 3000 equipment (Dionex). Peptides were loaded onto an Acclaim pepmap RSLC C18 column (Dionex) and eluted by a 2–45% acetonitrile linear gradient. Fractions were collected onto a 384 anchorchip MALDI plate via a Proteineer FcII fractionator (Bruker) and mixed with α-Cyano-4-hydroxycinnamic acid directly upon deposition. Sample acquisition in TOF and TOF/TOF modes was performed automatically on an Autoflex speed MALDI mass spectrometer (Bruker). Peptide assignments, protein identification and scoring were managed on a Proteinscape server (allowing a 50 ppm tolerance for mass measurements) through interrogation of the NCBI nr database on a local Mascot server.

### Plasmid pOri23-camp^515^ construction and transformation

Characteristics of the pOri23 plasmid [52] are listed in Table S1. Cloning steps were performed using *E. coli* as host strain. Chromosomal DNA from *S. agalactiae* 515 was prepared using DNAeasy Blood & Tissue Kit (Qiagen, Hilden, Germany) according to the manufacturer's instructions. The CAMP factor II encoding gene (SAL_2074), as well as its endogenous RBS and terminator sites, was amplified using the Phusion® High-Fidelity DNA Polymerase (Finnzymes, Keilaranta, Finland) and primers CAMP factor ICE_*515_tRNA^Lys^ Bam*HI fwd and CAMP factor ICE_*515_tRNA^Lys^ Nsi*I Rev (Table S2). *Bam*HI and *Nsi*I restriction sites were incorporated at the 5′ end of each primer in order to allow the final ligation. The 922 bp PCR fragment was verified on agarose gel and subcloned using Zero Blunt® TOPO® in chemically competent Top10 cells (In Vitrogen life Technologies, Carlsbad, USA). The obtained vector was extracted using NucleoSpin® Plasmid columns (Macherey-Nagel, Düren, Germany) and digested by *Bam*HI and *Nsi*I restriction enzymes (Promega, Madison, USA). The digested SAL_2074 insert was purified on NucleoSpin® Gel and PCR Clean-up columns (Macherey-Nagel, Düren, Germany). In parallel, native pOri23 was digested using *Bam*HI and *Pst*I enzymes, ligated to the SAL_2074 insert using T4 DNA ligase (Promega, Madison, USA) and transformed by electroporation into DH5α *E. coli* strain as described [49], [53]. Finally, the pOri23-camp^515^ was purified and electroporated into *L. lactis*, *S. bovis* and *S. dysgalactiae* strains as described previously [52], [54].

### PCR screening of ICE_*515_tRNA^Lys^* genes in strain collections

Chromosomal DNA from bacterial strains used in this study was prepared using DNAeasy Blood & Tissue Kit (Qiagen, Hilden, Germany) according to the manufacturer's instructions, and was used as template for DNA amplification. Standards PCR were performed in a final volume of 25 µL containing 1 U of Taq (Roche Applied Science, Mannheim, Germany), polymerase buffer 1×, 0.4 mM primers, 0.2 mM dNTP mix and 100 ng of DNA template. Primers used in this study are listed in Table S2. PCR conditions consisted in a first denaturation step at 95°C for 2 min followed by 30 cycles, each composed of a denaturation step at 95°C for 30 s, an annealing step at 5°C below primer's Tm for 30 s and an elongation step at 72°C for 1 min per kb with a final extension step of 10 min at 72°C. Sequencing reactions were performed by Beckman Coulter Genomics and data were treated using BioEdit free Software.

Long range PCRs were performed using GoTaq® Long PCR Master Mix (Promega, Madison, USA). PCR conditions consisted of a first denaturation step at 94°C for 2 min, followed by 20 cycles each composed of a denaturation step at 94°C for 30 s, an annealing step at 5°C below primer's Tm for 30 s and an elongation step at 65°C for 30 min, and followed by 15 cycles with an increment of 30 s at each elongation step. The final elongation step lasts 20 min at 72°C. 1 µL of the amplification products was used as template for nested PCR which were performed as standards PCR.

### CAMP reaction test

Strains were screened for CAMP activity as previously described [8]. Briefly, the beta-toxin producing strain *S. aureus* CIP 57.10 (Institut Pasteur, Paris) was streaked on 5% sheep blood agar Tryptic soy plates and strains to be tested were streaked perpendicularly to the first streak. After overnight incubation at 37°C, strains producing CAMP factor developed a characteristic arrow-shaped hemolysis.

### Hemolytic co-reaction monitoring

The co-hemolysis assays were performed using microtiter plates (NUNC immuno plate Maxisorb, Roskilde, Denmark) as previously described with minor modifications [55]. Briefly, a suspension of sheep erythrocytes (Biomérieux, Marcy l'étoile, France) was washed 5 times in hemolysis buffer (Tris-HCl, 10 mM; NaCl, 150 mM; pH 7.4) by centrifuging 30 min at 2000× *g* and then diluted in hemolysis buffer (1∶100). This suspension was treated with 0.025 U/mL of *S. aureus* sphingomyelinase (Sigma Aldrich, Steinheim, Germany) for 30 min at 37°C and then 100 µL were distributed in flat-bottomed wells. Bacterial suspensions were grown up to an OD_600_ of 0.8 and culture supernatants were collected by centrifugation 10 min at 2000× *g* and then0.2 µm-filtered. A 100 µL-volume of each supernatant was distributed in wells.

Co-hemolytic activity of CAMP factor II correlates with the loss of integrity of sheep erythrocytes visualized as a decrease in optical density measured at 630 nm (Biotek ELx808). In these experiments, controls were realized using *S. agalactiae* 515 strain as positive control and samples without sphingomyelinase treatment as negative controls.

## Results and Discussion

### ICE*_515_tRNA^Lys^*, a mobile genetic element found in 515 *S. agalactiae* invasive human strain, carries a putative new CAMP factor

**Figure 1:**
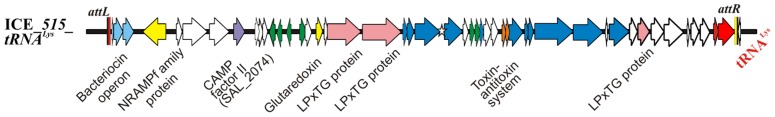
Schematic diagram of the ICE_*515_tRNA^Lys^* mobile genetic element. ORFs appear as arrows. Genes encoding the putative bacteriocin system appear in light blue. The genes encoding the proteins that could be involved in oxidative stress response (NRAMP protein and thioredoxin-like) are colored in yellow and the gene encoding the putative new hemolytic CAMP factor (CAMP factor II) in purple. Genes encoding the proteins with LPxTG motif appear in pink. Genes of the conjugation module are indicated with blue arrows and regulation module with green arrows. Putative *oriT* is indicated by a star. Genes encoding a putative toxin-antitoxin system appear in orange and other genes encoding proteins with unknown function in white. The gene where the element is integrated (tRNA^Lys^) is indicated in red. Recombination module is colored in red. Recombination sites are drawn as vertical rectangles. Black rectangles indicate identical sequences found in *attL*, *attR*, and *attI* sites; yellow rectangles indicate the arm of *attR* sites and the related arm of *attI* sites; and red rectangles indicate the arm of *attL sites* and the related arms of *attI* sites.

In addition to genes required for its mobility, maintenance or regulation, ICE_*515_tRNA^Lys^* also carries additional genes encoding proteins that can play a role in adaptation or virulence of the strain (Figure 1). BLASTp analyses suggest that these genes could encode (i) a putative bacteriocin system (SAL_2079 to SAL_2081 genes), (ii) 2 proteins that could be involved in oxidative stress response (SAL_2059 and SAL_2078 genes), (iii) 3 putative membrane proteins with a LPxTG motif (SAL_2036, SAL_2056 and SAL_2057), and (iv) a putative new hemolytic CAMP factor (CAMP factor II, SAL_2074 gene). Seven of eight GBS strains whose genome was sequenced carry an ICE or a related element at the 3′ extremity of the tRNA^Lys^ encoding gene. COHI, 2603 V/R and 18RS21 strains carry a putative ICE while NEM316, A909 and H36B strains carry a related element [33]. The element of COH1 strain carries the same accessory genes than strain 515, including CAMP factor II (SAN_2140, 100% of protein identity) but we recently showed that only ICE_*515_tRNA^Lys^* is able to self-transfer by conjugation (A. Puymege, submitted for publication). It is important to underline that the COH1 strain was used by Hensler *et al.* [15] to examine the contribution of CAMP factor to GBS systemic virulence. It cannot be excluded that this second CAMP factor confers a co-hemolytic activity in case of loss of the genomic CAMP factor. As a co-hemolysin, CAMP factor could contribute to erythrocyte lysis and thus to the release of haem which is one of the co-factors (with menaquinone) required for respiration growth of *S. agalactiae*. Respiration growth could be beneficial to GBS dissemination by increasing growth in blood [56]. Interestingly, an ICE with an integrase similar to those of ICE_*515_tRNA^Lys^* (87% of identity) was also found in *S. uberis* strain 19608 isolated from mastitis (S. Chuzeville, unpublished data).

### Characteristics, expression and production of CAMP factor II

**Figure 2:**
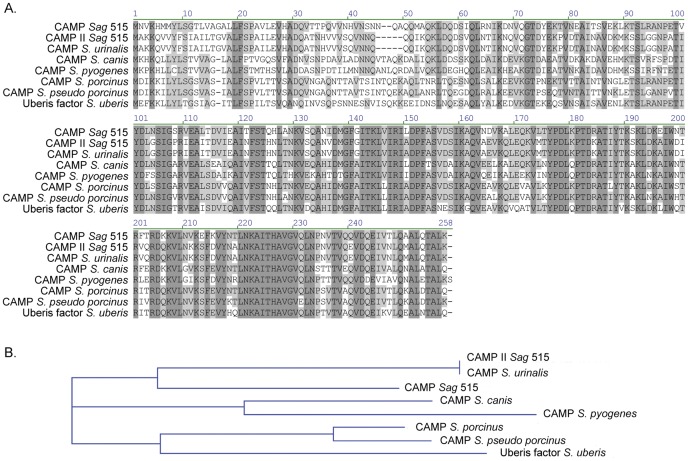
Comparison of CAMP factors and Uberis factor found in streptococci. A. multiple sequence alignment (*Sag*, *S. agalactiae*; *S. urinalis*; *S. canis*; *S. pyogenes*; *S. porcinus*; *S. pseudo porcinus* and *S. uberis*); B. phylogenetic tree showing the evolutionary relationships between the sequences of the alignment. Sequence alignment and construction of the phylogenetic tree were done using the AlignX module of VectorNTI advance 11 (InVitrogen). Conserved residues appear in light grey and identical amino acids appear in dark grey in the alignment. Position of residues in the sequence is indicated above the sequence. Sequence identities go from 56% (CAMP factor II of *S. agalactiae* and Uberis factor of *S. uberis*) to 100% (CAMP factor II of *S. agalactiae* and CAMP factor of *S. urinalis*). The CAMP factor of *P. acnes* is more distant (less than 30% of identity) and thus does not appear in this alignment. The phylogenetic tree has been constructed using the Neighbor Joining Method. Each branch of the tree has a length equal to the number of substitutions required to get from one nod to the next.

The genomic CAMP factor already described in *S. agalactiae* [8] is encoded by the SAL_2095 gene (accession number: ZP_00790029) in strain 515 and GBS2000 gene in strain NEM316 (accession number: NP_736433). SAL_2095 and GBS2000 genes encode identical proteins. BLASTp revealed 73% of identity between genomic CAMP factor compared to COHI and 515 CAMP factor II (Figure 2). Some of *S. uberis* strains produce a CAMP factor like named Uberis factor (accession number: AAA78910), which exhibits protein identity of 63% and 59% compared to SAL_2095 and SAL_2074, respectively.

Analysis of the SAL_2074 gene promoter region showed the presence of a Pribnow box (TATACT) located 17 pb upstream of a -35 box (TTGACA). A Ribosomal Binding Site (AGGAGG) is located 7 pb downstream of the SAL_2074 start codon. All sequence elements required for transcription and translation of the gene are thus present.

**Figure 3:**
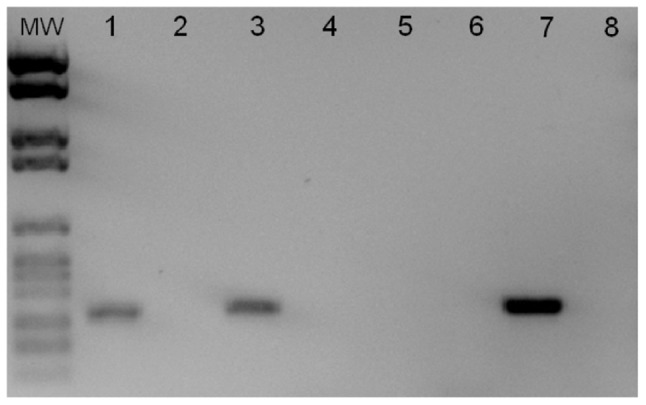
Analysis of the expression of the CAMP factor II (SAL_2074) gene. RT-PCR were performed using, as templates, RNA extracted from stationary-phase cultures of strain 515 (1, and negative control without RTase, 4); strain NEM316 (2 and negative control without RTase, 5); and transconjugant NEM316 (ICE_*515_tRNA^Lys^*) (3 and negative control without RTase, 6). The same results were obtained in exponential phase. A positive control using genomic DNA of strain 515 was included (7) as well as a negative control with water (8). DNA molecular weight marker is marker number VI (Roche Applied Science).

To check for gene transcription, RT-PCR were performed on 515, NEM316 and NEM316 (ICE_*515_tRNA^Lys^*) transconjugant strains. In strain 515, the CAMP factor II gene (SAL_2074) was expressed in exponential and stationary phases in the conditions tested. Transfer of ICE_*515_tRNA^Lys^* in strain NEM316 enables the expression of the SAL_2074 gene in this strain (Figure 3).

To examine production and secretion of CAMP factor II, a CAMP test was performed. All strains produced a characteristic arrow-shaped hemolysis on plates and no difference could be observed between NEM316 strains carrying ICE_*515_tRNA^Lys^* or not (Figure S1A).

Hemolysis monitoring was performed on these strains. This experiment also indicated a CAMP factor secretion by 515, NEM316 and NEM316 (ICE_*515_tRNA^Lys^*) strains (Figure S1B). However, the measured co-hemolytic activity appeared to reflect those of genomic CAMP factor as there was no additional activity in NEM316 (ICE_*515_tRNA^Lys^*) transconjugant (with both CAMP factors) compared to strain NEM316 (with only genomic CAMP factor). The quantity of CAMP factor II secreted in the supernatant was analyzed and compared with the one of CAMP factor by SDS-PAGE and liquid chromatography coupled with mass spectrometry. These experiments indicated that CAMP factor II is secreted in the supernatant at the same level than CAMP factor (Figure S2). Despite being abundantly secreted, CAMP factor II does not seem to confer additional co-hemolytic activity to a strain already expressing genomic CAMP factor.

Interestingly, we recently showed that ICE_*515_tRNA^Lys^* confers adhesive properties to recipient strain (S. Chuzeville, unpublished results) which could enable the maintenance of this ICE, also carrying the additional CAMP factor, in the GBS population.

### CAMP factor II can provide hemolytic functions to non-pathogenic strains

Several unsuccessful attempts were made to construct a mutant lacking the CAMP factor II encoding gene. This could be due to an intracellular replication of the element causing recombination problems during mutant construction [57]. To solve this problem and to study the CAMP factor II functionality, an expression vector was constructed, allowing the production of the protein of interest in a host strain.

**Figure 4:**
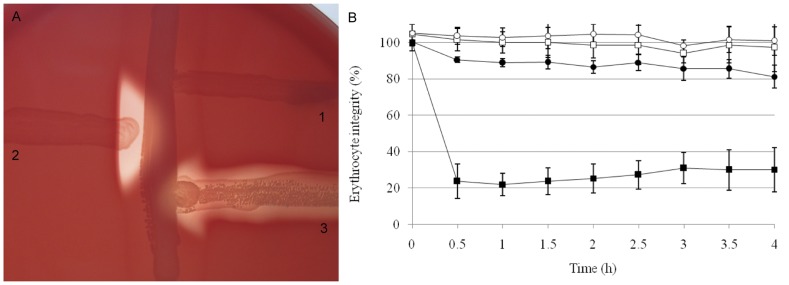
Co-hemolytic activity of CAMP factor II expressed in *L. lactis* MG1363. A. CAMP test using (1) *L. lactis* MG1363 (pOri23), (2) *L. lactis* MG1363 (pOri23-camp^515^) and (3) GBS strain 515 as control; B. *L. lactis* co-hemolytic activity was monitored in *L. lactis* MG1363 (pOri23) (filled circle with SMase treatment and empty circle without treatment) and in *L. lactis* MG1363 (pOri23-camp^515^) (filled squares with SMase treatment and empty squares without treatment). Hemolytic activity was measured at OD_630_ every 30 min using a microplate reader. The experiment was done in triplicate using three independent biological samples. Errors bars represent the standard deviation observed between the 9 values obtained for each strain. Controls without SMase treatment were done (data not shown).

The functionality of CAMP factor II was first analyzed using *L. lactis* MG1363 strain (Fig. 4). The SAL_2074 gene was expressed under the P23 constitutive promoter using the pOri23 vector. Expression was confirmed by RT-PCR (data not shown). A characteristic arrow-shaped hemolysis similar to that produced by 515 GBS strain was observed in *L. lactis* MG1363 (pOri23-camp^515^) compared with control *L. lactis* MG1363 (pOri23) strain (Figure 4A). The functionality of CAMP factor II was confirmed by a hemolytic co-reaction titration (Figure 4B).

We thus showed that CAMP factor II can be expressed, produced and secreted correctly in a non-pathogenic species. ICE_*515_tRNA^Lys^* can autonomously transfer to other *S. agalactiae* strains (A. Puymege, submitted for publication) so this element can spread this pore-forming toxin-encoding gene in the GBS population. Since interspecies transfer likely occurs in natural habitats, as similar elements have been detected in *S. uberis* and *S. dysgalactiae* strains [38], CAMP factor II may confer hemolytic properties if ICE_*515_tRNA^Lys^* was transferred to CAMP-negative recipient strains.

### CAMP factor II is also present in other pathogenic streptococcal strains of veterinary origin

A collection of streptococcal pathogenic strains of veterinary origin was studied to test the ability of the strains to generate a co-haemolytic reaction with *S. aureus*. This collection included isolates of *S. uberis* (n = 449); *S. dysgalactiae* (n = 162); *S. bovis* (n = 31); *S. suis* (n = 26) and *S. equisimilis* (n = 9). Twenty-three strains were CAMP positive: 20 *S. uberis* (4%), 1 *S. bovis* (3%), 1 *S. equisimilis* (11%) and 1 *S. dysgalactiae* (less than 1%) strain. CAMP-positive strains were collected in 11 different geographic areas throughout France between 1984 and 2010.

Detection of the gene encoding the factor responsible for co-hemolytic reaction in the streptococcal collection was performed by amplification using primers designed from CAMP factor II and Uberis factor gene sequences. Only the factor responsible for the reaction observed for *S. dysgalactiae* strain 24084 could not be characterized in this study. Sequencing of the co-hemolytic factor encoding gene in *S. uberis* strains revealed that all the strains tested (n = 10) carried an Uberis factor-like gene (identity>97%), whereas *S. bovis* and *S. equisimilis* carried a CAMP factor gene very similar to the one carried by ICE_*515_tRNA^Lys^* (96% of identity for both).

**Figure 5:**
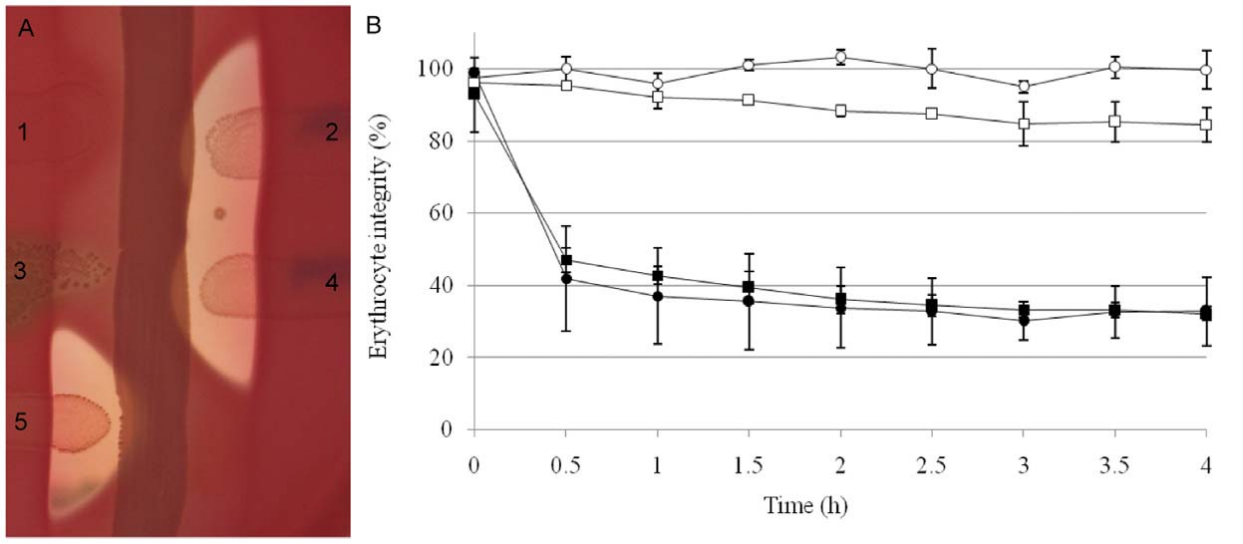
Expression of CAMP factor II in pathogenic streptococcal strains. A. CAMP test using (1) *S. bovis* 1052 wt, (2) *S. bovis* (pOri23-camp^515^), (3) *S. dysgalactiae* 593 wt, (4) *S. dysgalactiae* (pOri23-camp^515^) and (5) GBS strain 515 as control; B. co-hemolytic activity was monitored in *S. bovis* 1052 (filled circle with pOri23-camp^515^ and empty circle without) and in *S. dysgalactiae* (filled squares with pOri23-camp^515^ and empty squares without treatment). Hemolytic activity was measured at OD_630_ every 30 min using a microplate reader. The experiment was done in triplicate using three independent biological samples. Errors bars represent the standard deviation observed between the 9 values obtained for each strain. Controls without SMase treatment were carried out (data not shown).

CAMP factors have already been described in other streptococci such as *S. pyogenes* [20], [58], *S. uberis* [14], [22], [59], [60], *S. porcinus* [22], [59], [61], [62], *S. canis* [22], [23], [62], *S. intestinalis* [63]. Furthermore BLASTp analysis showed that *cfb* homologues in *P. acnes*, *S. uberis*, *S. canis, S. porcinus* and *S. pyogenes* exhibit significant homologies with CAMP factor (34 to 99%). However, this is the first time to our knowledge that a CAMP factor is described in *S. bovis* and in *S. equisimilis*. CAMP-negative field strains were examined for their ability to express CAMP factor II. For this purpose, pOri23-camp^515^ was transformed in two different field strains: one *S. bovis* strain and one *S. dysgalactiae* strain. A characteristic arrow-shaped hemolysis similar to that produced by 515 GBS strain was observed when CAMP factor II was expressed in field strains (Fig. 5A) when compared with wild strains. This result was confirmed by hemolytic co-reaction titration (Fig. 5B).

Collectively, these results confirm that the CAMP factor II is not primarily a characteristic of GBS and enhance our belief that the routine detection of GBS by usual CAMP test on plates or using PCR methods should always be confirmed by other phenotypic or genotypic methods, since the CAMP factor II or Uberis factor present in a large range of streptococci might lead to a misidentification.

Nevertheless, no *S. suis* strain in the tested collection carried functional *cfb* homologues. This is consistent with previous work that showed that genetic exchange between *S. suis* and other species appeared to be uncommon [36].

Finally, no previous work has shown the presence of such co hemolytic factors in *S. bovis* and *S. equisimilis* and the similarity between CAMP factor II and those of these two field strains suggests a recent acquisition by horizontal transfer probably mediated by ICE carrying this gene.

### CAMP positive strains carry a genetic element integrated at the 3′ end of tRNA^Lys^ gene

CAMP-positive strains were analyzed for the presence of a genetic element integrated in the tRNA^Lys^ (with CTT anticodon) encoding gene. First, ICE_*515_tRNA^Lys^* and ICE_*SUB19608_tRNA^Lys^* integrase genes were searched and, then, intergenic regions between integrase and tRNA^Lys^ gene and more precisely the *attR* ICE_*515_tRNA^Lys^* integration specific site were amplified (Table S2).

Twenty one of the 23 strains tested, including *S. bovis* 7434, *S. equisimilis* 20591 and *S. dysgalactiae* 24084 strains, showed an integrase gene similar to that of ICE_*SUB19608_tRNA^Lys^*.

*attR* sites were searched using primers ICE_*SUB19608_tRNA^Lys^* fwd and tRNA^Lys^ AAA Rev (Table S2). An *attR* site was amplified for 17 of the 23 strains tested including *S. bovis* 7434 and *S. equisimilis* 20591. PCR products were then confirmed by sequencing which revealed that the *attR* site of these strains was very close to that of ICE_*515_tRNA^Lys^* for 15 on 18 *S. uberis*, *S. bovis* 7434 and *S. equisimilis* 20591 (78 to 86% of identity) (see Fig. S3).

Long range PCRs were performed to examine the position of CAMP factor II encoding gene on *S. bovis* and *S. equisimilis* strains. PCRs, made using primers designed from ICE_*515_tRNA^Lys^* and located in CAMP factor II encoding gene and tRNA^Lys^ gene, gave negative results in *S. bovis* and *S. equisimilis* strains. In strain 515, this region has a length of more than 34 kb which could explain the difficulty to amplify it in field strains. However, amplicons larger than 10 kb were obtained for both field strains using one primer hybridizing to the CAMP factor II encoding gene (CAMP factor ICE_*515_tRNA^Lys^ Nsi*I Rev for both) and the second primer hybridizing to the gene upstream of the putative mobile genetic element (HMPREF9319_0116 fwd for *S. bovis* strain and HMPREF9964_2030 fwd for *S. equisimilis* strain) (Table S2). In strain 515, this region has also a length of about 10 kb. To confirm that the amplicon corresponds to the expected region, nested PCRs were performed using long range PCR products as templates and primers hybridizing to CAMP factor II encoding gene (CAMP factor ICE_*515_tRNA^Lys^ Bam*HI fwd and CAMP factor ICE_*515_tRNA^Lys^* Rev) (Table S2).

Thus, CAMP factor II encoding genes in *S. bovis* and *S. equisimilis* strains were located on a genetic element integrated at the 3′ end of the tRNA^Lys^ encoding gene.

Hence two thirds of the strains which produce a CAMP factor II appeared to carry an element integrated at 3′ end of tRNA^Lys^ gene. This suggests that the spread of the CAMP factor II gene in different pathogenic streptococcal species is linked to the transfer of an ICE carrying this gene.

## Conclusions

In conclusion, the SAL_2074 gene carried by a mobile genetic element (ICE_*515_tRNA^Lys^*) in *S. agalactiae* 515 strain encodes a functional CAMP factor. An identical CAMP factor is encoded by a putative ICE carried by *S. agalactiae* COH1. It is important to mention that this strain has been used by Hensler *et al.* to conclude that CAMP factor is not essential for GBS virulence [15]. It is possible that this second CAMP factor confers co-hemolysin activity in case of loss of the genomic CAMP factor. A similar toxin is also encoded by a putative ICE detected in *S. urinalis* although this strain gave a negative result using *in vitro* CAMP test [64]. This toxin is functional when expressed in non pathogenic (*L. lactis*) and other pathogenic streptococcal species (*S. dysgalactiae*, *S. bovis*). Furthermore, screening of a collection of strains of different streptococcal species indicated that some are positive for the CAMP test. All strains (except *S. uberis* 21459 and 22492 strains in the conditions used) also carry the integrase gene of ICE_*515_tRNA^Lys^* thus suggesting a spread of the CAMP factor II gene among different streptococcal species through ICE conjugative transfer.

## Supporting Information

Figure S1 **Co-hemolytic activity of CAMP factor II in GBS strains.** A. CAMP test using (1) NEM316, (2) NEM316 (ICE_*515_tRNA^Lys^*), and (3) 515 GBS strains; B. Measure of co-hemolytic activity of CAMP factor(s) in *S. agalactiae* strain 515 (filled circles with SMase treatment and empty circle without treatment), strain NEM316 (filled triangles with SMase treatment and empty triangles without treatment) and transconjugant NEM316 (ICE_*515_tRNA^Lys^*) (filled squares with SMase treatment and empty squares without treatment). Hemolytic activity was measured at OD_630_ every 30 min using a microplate reader. The experiment was done in triplicate using three independent biological samples. Errors bars represent the standard deviation observed between the 9 values obtained for each strain.
(TIF)

Figure S2 **SDS-PAGE analysis of the supernant of** ***S. agalactiae*** **NEM316 and NEM316 (ICE_** ***515_tRNA^Lys^*** **) cultures.** Supernatant of overnight cultures of *S. agalactiae* NEM316 (1, in BHI and 3, in CDM) and of *S. agalactiae* NEM316 (ICE_*515_tRNA^Lys^*) (2, in BHI and 4, in CDM). Molecular weight marker (MW) is unstained protein MW marker of Euromedex. MWs are indicated on the right side of the gel. Arrows indicated the position of the band corresponding to genomic CAMP factor (I) and CAMP factor II (II).
(TIF)

Figure S3 **Alignment of** ***attR*** **sequence of ICE_** ***515_tRNA^Lys^*** **in field strain.** Sequence alignment was done using the AlignX module of VectorNTI advance 11 (InVitrogen).
(TIF)

Table S1 **Strains and their characteristics.** Strains and plasmids are listed together with their associated pathologies or genotypic characteristics, the year and geographic place of isolation, their hemolysis pattern, the CAMP test results and the reference for strains or plasmids.
(DOC)

Table S2 **Primers used in this work.** Restriction sites appear in bold.
(DOC)

## References

[1] Farley MM (2001) Group B streptococcal disease in nonpregnant adults. Clin Infect Dis 33: 556–561. 11462195 10.1086/322696

[2] Martins ER, Florindo C, Martins F, Aldir I, Borrego MJ, et al (2007) *Streptococcus agalactiae* serotype Ib as an agent of meningitis in two adult nonpregnant women. J Clin Microbiol 45: 3850–3852. 17881554 10.1128/JCM.01358-07 PMC2168519

[3] Cordoba-Lopez A, Bueno Alvarez-Arenas MI, Monterrubio-Villa J, Corcho-Sanchez G (2002) *Streptococcus agalactiae* pleural empyema in a healthy adult. Enferm Infecc Microbiol Clin 20: 478–479. 12425886 10.1016/s0213-005x(02)72848-6

[4] Keefe GP (1997) *Streptococcus agalactiae* mastitis: a review. Can Vet J 38: 429–437. 9220132 PMC1576741

[5] Rajagopal L (2009) Understanding the regulation of Group B Streptococcal virulence factors. Future Microbiol 4: 201–221. 19257847 10.2217/17460913.4.2.201 PMC2691590

[6] Maisey HC, Doran KS, Nizet V (2008) Recent advances in understanding the molecular basis of group B Streptococcus virulence. Expert Rev Mol Med 10: e27. 18803886 10.1017/S1462399408000811 PMC2676346

[7] Lang S, Palmer M (2003) Characterization of *Streptococcus agalactiae* CAMP factor as a pore-forming toxin. J Biol Chem 278: 38167–38173. 12835325 10.1074/jbc.M303544200

[8] Christie R (1944) A note on a lytic phenomenon shown by group B streptococci. Aust J Exp Biol 22: 197–200. 10.1038/icb.1945.30 21006102

[9] Milhas D, Clarke CJ, Hannun YA (2010) Sphingomyelin metabolism at the plasma membrane: implications for bioactive sphingolipids. FEBS Lett 584: 1887–1894. 19857494 10.1016/j.febslet.2009.10.058 PMC2856805

[10] Titball RW (1993) Bacterial phospholipases C. Microbiol Rev 57: 347–366. 8336671 10.1128/mr.57.2.347-366.1993 PMC372913

[11] Rodi PM, Cabeza MS, Gennaro AM (2006) Detergent solubilization of bovine erythrocytes. Comparison between the insoluble material and the intact membrane. Biophys Chem 122: 114–122. 16580771 10.1016/j.bpc.2006.03.005

[12] Lang S, Xue J, Guo Z, Palmer M (2007) *Streptococcus agalactiae* CAMP factor binds to GPI-anchored proteins. Med Microbiol Immunol 196: 1–10. 16773378 10.1007/s00430-006-0021-2

[13] Jürgens D (1987) Unspecific binding of group B streptococcal co-cytolysin (CAMP factor) to immunoglobulins and its possible role in pathogenicity. J Exp Med 165: 730–732. 10.1084/jem.165.3.720 PMC2188285 3546580

[14] Skalka B, Smola J (1981) Lethal effect of CAMP-factor and UBERIS-factor: a new finding about diffusible exosubstances of *streptococcus agalactiae* and *Streptococcus uberis* . Zentralbl Bakteriol A 249: 190–194. 7023125

[15] Hensler ME, Quach D, Hsieh CJ, Doran KS, Nizet V (2008) CAMP factor is not essential for systemic virulence of Group B Streptococcus. Microb Pathog 44: 84–88. 17870297 10.1016/j.micpath.2007.08.005 PMC2247369

[16] Fuchs PC, Christy C, Jones RN (1978) Multiple-inocula (replicator) CAMP test for presumptive identification of group B streptococci. J Clin Microbiol 7: 232–233. 344342 10.1128/jcm.7.2.232-233.1978 PMC274897

[17] Phillips EA, Tapsall JW, Smith DD (1980) Rapid tube CAMP test for identification of *Streptococcus agalactiae* (Lancefield group B). J Clin Microbiol 12: 135–137. 7014603 10.1128/jcm.12.2.135-137.1980 PMC273541

[18] Ke D, Menard C, Picard FJ, Boissinot M, Ouellette M, et al (2000) Development of conventional and real-time PCR assays for the rapid detection of group B streptococci. Clin Chem 46: 324–331. 10702518

[19] Ratner HB, Weeks LS, Stratton CW (1986) Evaluation of spot CAMP test for identification of group B streptococci. J Clin Microbiol 24: 296–297. 3528214 10.1128/jcm.24.2.296-297.1986 PMC268893

[20] Gase K, Ferretti JJ, Primeaux C, McShan WM (1999) Identification, cloning, and expression of the CAMP factor gene (*cfa*) of group A streptococci. Infect Immun 67: 4725–4731. 10456923 10.1128/iai.67.9.4725-4731.1999 PMC96801

[21] Jiang M, Babiuk LA, Potter AA (1996) Cloning, sequencing and expression of the CAMP factor gene of *Streptococcus uberis* . Microb Pathog 20: 297–307. 9132527 10.1006/mpat.1996.0028

[22] Hassan AA, Abdulmawjood A, Yildirim AO, Fink K, Lammler C, et al (2000) Identification of streptococci isolated from various sources by determination of *cfb* gene and other CAMP-factor genes. Can J Microbiol 46: 946–951. 11068682

[23] Gurturk K, Lammler C (1990) Purification and partial characterization of a co-haemolysin (CAMP-factor) produced by *Streptococcus canis* . FEMS Microbiol Immunol 2: 97–102. 2257165 10.1111/j.1574-6968.1990.tb03506.x

[24] Valanne S, McDowell A, Ramage G, Tunney MM, Einarsson GG, et al (2005) CAMP factor homologues in *Propionibacterium acnes*: a new protein family differentially expressed by types I and II. Microbiology 151: 1369–1379. 15870447 10.1099/mic.0.27788-0

[25] Doolittle WF (1999) Lateral genomics. Trends Cell Biol 9: 5–8. 10611671

[26] de la Cruz F, Davies J (2000) Horizontal gene transfer and the origin of species: lessons from bacteria. Trends Microbiol 8: 128–133. 10707066 10.1016/s0966-842x(00)01703-0

[27] Kurland CG (2000) Something for everyone. Horizontal gene transfer in evolution. EMBO Rep 1: 92–95. 11265763 10.1093/embo-reports/kvd042 PMC1084273

[28] Lawrence JG, Ochman H (1998) Molecular archaeology of the *Escherichia coli* genome. Proc Natl Acad Sci U S A 95: 9413–9417. 9689094 10.1073/pnas.95.16.9413 PMC21352

[29] Burrus V, Pavlovic G, Decaris B, Guedon G (2002) Conjugative transposons: the tip of the iceberg. Mol Microbiol 46: 601–610. 12410819 10.1046/j.1365-2958.2002.03191.x

[30] Wozniak RA, Waldor MK (2010) Integrative and conjugative elements: mosaic mobile genetic elements enabling dynamic lateral gene flow. Nat Rev Microbiol 8: 552–563. 20601965 10.1038/nrmicro2382

[31] Burrus V, Waldor MK (2004) Shaping bacterial genomes with integrative and conjugative elements. Res Microbiol 155: 376–386. 15207870 10.1016/j.resmic.2004.01.012

[32] Tettelin H, Masignani V, Cieslewicz MJ, Donati C, Medini D, et al (2005) Genome analysis of multiple pathogenic isolates of *Streptococcus agalactiae*: implications for the microbial “pan-genome”. Proc Natl Acad Sci U S A 102: 13950–13955. 16172379 10.1073/pnas.0506758102 PMC1216834

[33] Brochet M, Couve E, Glaser P, Guedon G, Payot S (2008) Integrative conjugative elements and related elements are major contributors to the genome diversity of *Streptococcus agalactiae* . J Bacteriol 190: 6913–6917. 18708498 10.1128/JB.00824-08 PMC2566197

[34] Beres SB, Musser JM (2007) Contribution of exogenous genetic elements to the group A Streptococcus metagenome. PLoS One 2: e800. 17726530 10.1371/journal.pone.0000800 PMC1949102

[35] Croucher NJ, Walker D, Romero P, Lennard N, Paterson GK, et al (2009) Role of conjugative elements in the evolution of the multidrug-resistant pandemic clone *Streptococcus pneumoniae* Spain23F ST81. J Bacteriol 191: 1480–1489. 19114491 10.1128/JB.01343-08 PMC2648205

[36] Holden MT, Hauser H, Sanders M, Ngo TH, Cherevach I, et al (2009) Rapid evolution of virulence and drug resistance in the emerging zoonotic pathogen *Streptococcus suis* . PLoS One 4: e6072. 19603075 10.1371/journal.pone.0006072 PMC2705793

[37] Ward PN, Holden MT, Leigh JA, Lennard N, Bignell A, et al (2009) Evidence for niche adaptation in the genome of the bovine pathogen *Streptococcus uberis* . BMC Genomics 10: 54. 19175920 10.1186/1471-2164-10-54 PMC2657157

[38] Haenni M, Saras E, Bertin S, Leblond P, Madec JY, et al (2010) Diversity and mobility of integrative and conjugative elements in bovine isolates of *Streptococcus agalactiae*, *S. dysgalactiae* subsp. *dysgalactiae*, and *S. uberis* . Appl Environ Microbiol 76: 7957–7965. 20952646 10.1128/AEM.00805-10 PMC3008251

[39] Richards VP, Lang P, Bitar PD, Lefebure T, Schukken YH, et al (2011) Comparative genomics and the role of lateral gene transfer in the evolution of bovine adapted *Streptococcus agalactiae* . Infect Genet Evol 11: 1263–1275. 21536150 10.1016/j.meegid.2011.04.019 PMC3139733

[40] Manning SD, Springman AC, Million AD, Milton NR, McNamara SE, et al (2010) Association of Group B Streptococcus colonization and bovine exposure: a prospective cross-sectional cohort study. PLoS One 5: e8795. 20098699 10.1371/journal.pone.0008795 PMC2808344

[41] Bellanger X, Roberts AP, Morel C, Choulet F, Pavlovic G, et al (2009) Conjugative transfer of the integrative conjugative elements ICE*St1* and ICE*St3* from *Streptococcus thermophilus* . J Bacteriol 191: 2764–2775. 19181800 10.1128/JB.01412-08 PMC2668402

[42] Davies MR, Shera J, Van Domselaar GH, Sriprakash KS, McMillan DJ (2009) A novel integrative conjugative element mediates genetic transfer from group G Streptococcus to other beta-hemolytic Streptococci. J Bacteriol 191: 2257–2265. 19168609 10.1128/JB.01624-08 PMC2655516

[43] Suzuki H, Lefebure T, Hubisz MJ, Pavinski Bitar P, Lang P, et al (2011) Comparative genomic analysis of the *Streptococcus dysgalactiae* species group: gene content, molecular adaptation, and promoter evolution. Genome Biol Evol 3: 168–185. 21282711 10.1093/gbe/evr006 PMC3056289

[44] Zhang S, Green NM, Sitkiewicz I, Lefebvre RB, Musser JM (2006) Identification and characterization of an antigen I/II family protein produced by group A Streptococcus. Infect Immun 74: 4200–4213. 16790795 10.1128/IAI.00493-06 PMC1489706

[45] Sitkiewicz I, Green NM, Guo N, Mereghetti L, Musser JM (2011) Lateral gene transfer of streptococcal ICE element RD2 (region of difference 2) encoding secreted proteins. BMC Microbiol 11: 65. 21457552 10.1186/1471-2180-11-65 PMC3083328

[46] Glaser P, Rusniok C, Buchrieser C, Chevalier F, Frangeul L, et al (2002) Genome sequence of *Streptococcus agalactiae*, a pathogen causing invasive neonatal disease. Mol Microbiol 45: 1499–1513. 12354221 10.1046/j.1365-2958.2002.03126.x

[47] Maguin E, Duwat P, Hege T, Ehrlich D, Gruss A (1992) New thermosensitive plasmid for gram-positive bacteria. J Bacteriol 174: 5633–5638. 1324906 10.1128/jb.174.17.5633-5638.1992 PMC206509

[48] Framson PE, Nittayajarn A, Merry J, Youngman P, Rubens CE (1997) New genetic techniques for group B streptococci: high-efficiency transformation, maintenance of temperature-sensitive pWV01 plasmids, and mutagenesis with Tn*917* . Appl Environ Microbiol 63: 3539–3547. 9293004 10.1128/aem.63.9.3539-3547.1997 PMC168659

[49] Sambrook J, Fritsch EF, Maniatis T (1989) Molecular cloning: a laboratory manual. Cold Spring Harbor Laboratory

[50] Letort C, Juillard V (2001) Development of a minimal chemically-defined medium for the exponential growth of *Streptococcus thermophilus* . J Appl Microbiol 91: 1023–1029. 11851809 10.1046/j.1365-2672.2001.01469.x

[51] Bensadoun A, Weinstein D (1976) Assay of proteins in the presence of interfering materials. Anal Biochem 70: 241–250. 1259145 10.1016/s0003-2697(76)80064-4

[52] Que YA, Haefliger JA, Francioli P, Moreillon P (2000) Expression of *Staphylococcus aureus* clumping factor A in *Lactococcus lactis* subsp. *cremoris* using a new shuttle vector. Infect Immun 68: 3516–3522. 10816506 10.1128/iai.68.6.3516-3522.2000 PMC97637

[53] Hanahan D (1983) Studies on transformation of *Escherichia coli* with plasmids. J Mol Biol 166: 557–580. 6345791 10.1016/s0022-2836(83)80284-8

[54] Buckley ND, Vadeboncoeur C, LeBlanc DJ, Lee LN, Frenette M (1999) An effective strategy, applicable to *Streptococcus salivarius* and related bacteria, to enhance or confer electroporation competence. Appl Environ Microbiol 65: 3800–3804. 10473378 10.1128/aem.65.9.3800-3804.1999 PMC99703

[55] Sorensen M, Mak TN, Hurwitz R, Ogilvie LA, Mollenkopf HJ, et al (2010) Mutagenesis of Propionibacterium acnes and analysis of two CAMP factor knock-out mutants. J Microbiol Methods 83: 211–216. 20850482 10.1016/j.mimet.2010.09.008

[56] Yamamoto Y, Poyart C, Trieu-Cuot P, Lamberet G, Gruss A, et al (2005) Respiration metabolism of Group B Streptococcus is activated by environmental haem and quinone and contributes to virulence. Mol Microbiol 56: 525–534. 15813741 10.1111/j.1365-2958.2005.04555.x

[57] Carraro N, Libante V, Morel C, Decaris B, Charron-Bourgoin F, et al (2011) Differential regulation of two closely related integrative and conjugative elements from *Streptococcus thermophilus* . BMC Microbiol 11: 238. 22024428 10.1186/1471-2180-11-238 PMC3234194

[58] Gubash SM (1978) Synergistic hemolysis phenomenon shown by an alpha-toxin-producing *Clostridium perfingens* and streptococcal CAMP factor in presumptive streptococcal grouping. J Clin Microbiol 8: 480–488. 215600 10.1128/jcm.8.5.480-488.1978 PMC275283

[59] Lammler C, Blobel H (1987) Synergistic and antagonistic hemolytic reactions of bacterial proteins. Berl Munch Tierarztl Wochenschr 100: 95–99. 3034233

[60] Lopes MF, Merquior VL, Peralta JM, Teixeira LM (1995) Partial characterization of the cohemolytic factor produced by *Streptococcus uberis* and comparison with the CAMP-factor. FEMS Immunol Med Microbiol 12: 205–212. 8745004 10.1111/j.1574-695X.1995.tb00193.x

[61] Thal E, Obiger G (1969) The CAMP phenomenon of *Streptococcus agalactiae* and new serologic Streptococcus group “U” and other bacteria types. Berl Munch Tierarztl Wochenschr 82: 126–130. 4899658

[62] Lammler C, Gedek W, Blobel H (1987) CAMP like reactions for presumptive identification of *Bacillus cereus* from bovines. Zentralbl Veterinarmed B 34: 395–396. 3120441 10.1111/j.1439-0450.1987.tb00413.x

[63] Soedarmanto I, Lammler C (1996) Comparative studies on streptococci of serological group G isolated from various origins. Zentralbl Veterinarmed B 43: 513–523. 8976617 10.1111/j.1439-0450.1996.tb00349.x

[64] Collins MD, Hutson RA, Falsen E, Nikolaitchouk N, LaClaire L, et al (2000) An unusual Streptococcus from human urine, *Streptococcus urinalis* sp. nov. Int J Syst Evol Microbiol 50 Pt 3: 1173–1178. 10843060 10.1099/00207713-50-3-1173

