# Supplementary figures and images for: Characterization of a New CAMP Factor Carried by an Integrative and Conjugative Element in Streptococcus agalactiae and Spreading in Streptococci
Source: PLoS One. 2012 Nov 9;7(11):e48918. doi: 10.1371/journal.pone.0048918 (PMC3494709; doi:10.1371/journal.pone.0048918)

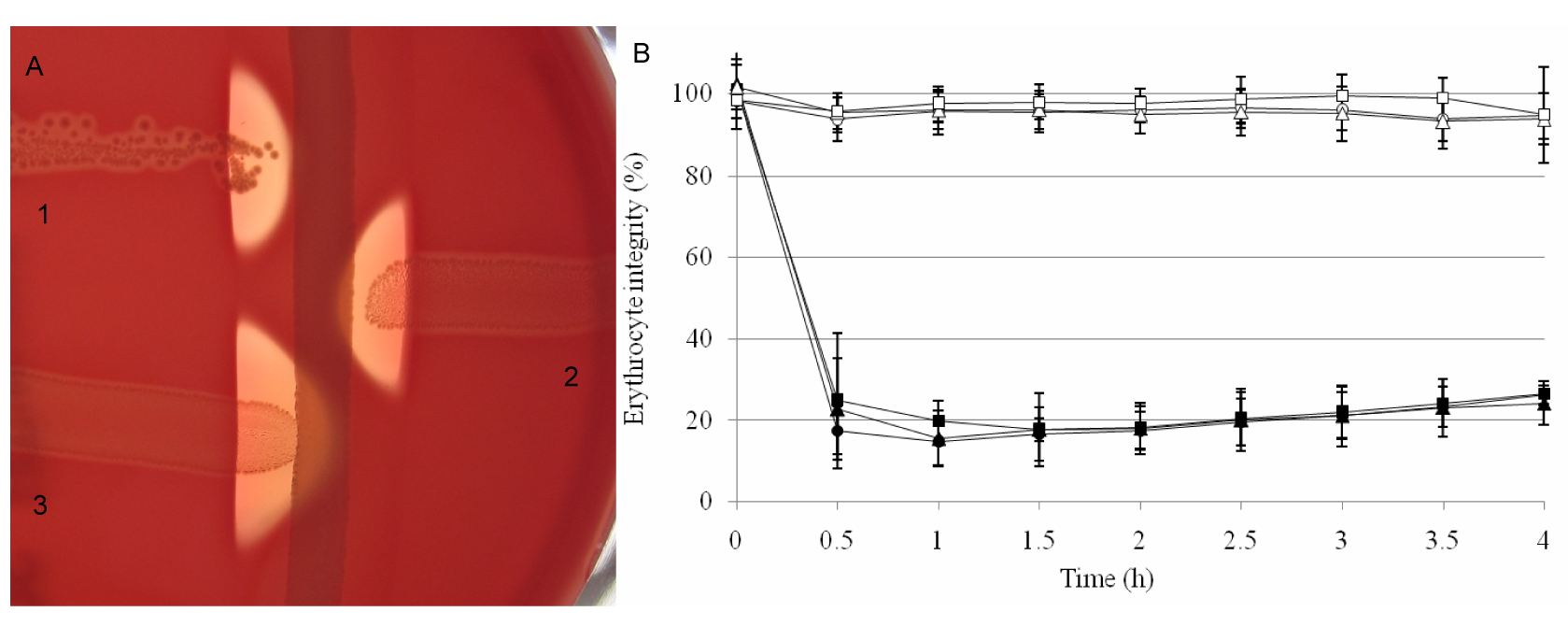

Supplement: Figure S1 — Co-hemolytic activity of CAMP factor II in GBS strains. A. CAMP test using (1) NEM316, (2) NEM316 (ICE_515_tRNALys), and (3) 515 GBS strains; B. Measure of co-hemolytic activity of CAMP factor(s) in S. agalactiae strain 515 (filled circles with SMase treatment and empty circle without treatment), strain NEM316 (filled triangles with SMase treatment and empty triangles without treatment) and transconjugant NEM316 (ICE_515_tRNALys) (filled squares with SMase treatment and empty squares without treatment). Hemolytic activity was measured at OD630 every 30 min using a microplate reader. The experiment was done in triplicate using three independent biological samples. Errors bars represent the standard deviation observed between the 9 values obtained for each strain. (TIF) [file pone.0048918.s001.tif]

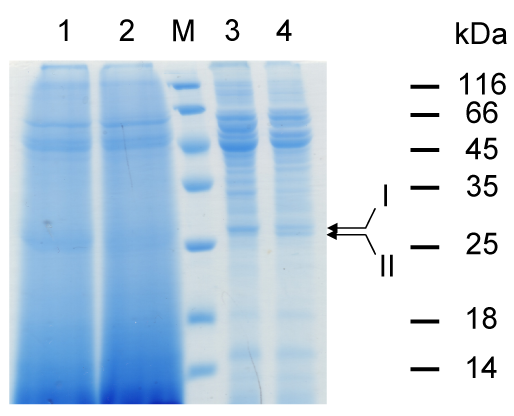

Supplement: Figure S2 — SDS-PAGE analysis of the supernant of S. agalactiae NEM316 and NEM316 (ICE_ 515_tRNALys ) cultures. Supernatant of overnight cultures of S. agalactiae NEM316 (1, in BHI and 3, in CDM) and of S. agalactiae NEM316 (ICE_515_tRNALys) (2, in BHI and 4, in CDM). Molecular weight marker (MW) is unstained protein MW marker of Euromedex. MWs are indicated on the right side of the gel. Arrows indicated the position of the band corresponding to genomic CAMP factor (I) and CAMP factor II (II). (TIF) [file pone.0048918.s002.tif]

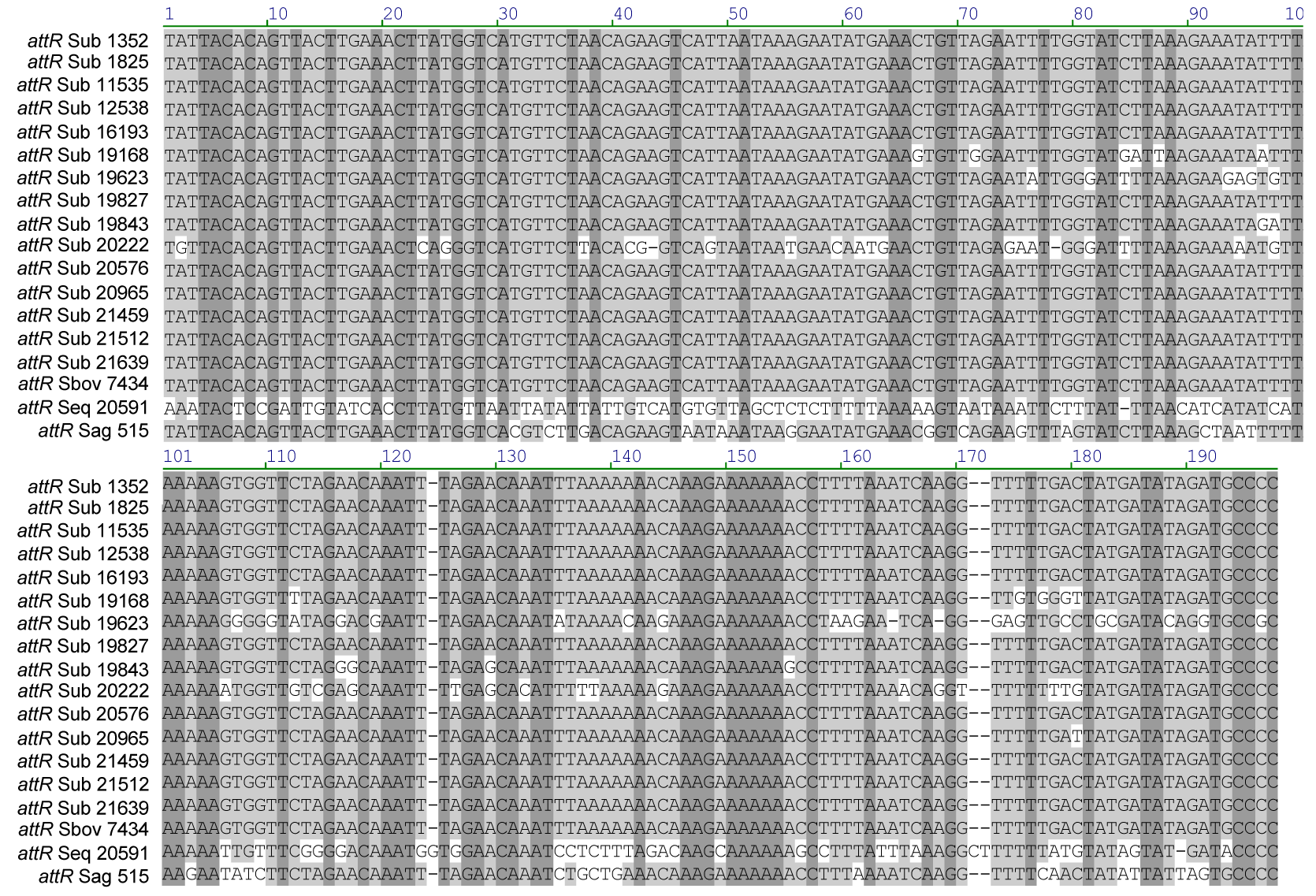

Supplement: Figure S3 — Alignment of attR sequence of ICE_ 515_tRNALys in field strain. Sequence alignment was done using the AlignX module of VectorNTI advance 11 (InVitrogen). (TIF) [file pone.0048918.s003.tif]
