# Supplementary material for: Characterization of a New CAMP Factor Carried by an Integrative and Conjugative Element in Streptococcus agalactiae and Spreading in Streptococci
Source: PLoS One. 2012 Nov 9;7(11):e48918. doi: 10.1371/journal.pone.0048918 (PMC3494709; doi:10.1371/journal.pone.0048918)
Supplement: Table S1 — Strains and their characteristics. Strains and plasmids are listed together with their associated pathologies or genotypic characteristics, the year and geographic place of isolation, their hemolysis pattern, the CAMP test results and the reference for strains or plasmids. (DOC) [file pone.0048918.s004.doc]

**Supporting Information Table 1. Strains and their characteristics.**

Strains and plasmids are listed together with their associated pathologies or genotype characteristics, the year and geographic place of isolation, their hemolysis pattern, the CAMP test results and the reference for strains or plasmids.

| **Species** | **Strain or plasmids** | **Pathology or relevant genotype** | **Sampling year** | **Geographic origin** | **Hemolysis** | **CAMP reaction** | **Source or reference** |
| --- | --- | --- | --- | --- | --- | --- | --- |
| *S. agalactiae* | 515 (ATCC BAA-1177) | Invasive | / | / | β | **+** | LGC standards |
|  | NEM316 | Septicaemia | / | / | β | **+** | [46] |
|  | NEM316 (ICE_*515_tRNA^Lys^*) | / | / | / | β | **+** | Puymege, submitted |
| *E. coli* | DH5α | General cloning | / | / | γ | **-** | [53] |
|  | Top10 | General cloning | / | / | γ | **-** | In Vitrogen, USA |
| *L. lactis* subsp. *cremoris* | MG1363 | / | / | / | γ | **-** | [52] |
|  | MG1363 (pOri23-camp^515^) | / | / | / | γ | **+** | This work |
| *S. aureus* | CIP57.10 | Reference CAMP reaction strain | / | / | β | **/** | Institut Pasteur, Paris |
| *S. uberis* | 21639 | Mastitis | / | / | γ | + | Resapath |
|  | 21457 | Mastitis | 2008 | 76 | α | + | Resapath |
|  | 21459 | Mastitis | 2008 | 76 | α | + | Resapath |
|  | 21468 | Mastitis | 2008 | 76 | α | + | Resapath |
|  | 21512 | Mastitis | 2008 | 61 | γ | + | Resapath |
|  | 22492 | Mastitis | 2009 | 82 | γ | + | Resapath |
|  | 19827 | Mastitis | 1985 | 64 | γ | + | Resapath |
|  | 19843 | Mastitis | 1984 | 22 | γ | + | Resapath |
|  | 19908 | / | 1984 | 22 | γ | + | Resapath |
|  | 19909 | Mastitis | 1985 | 22 | γ | + | Resapath |
|  | 1352 | Mastitis | 1986 | 22 | γ | + | Resapath |
|  | 1825 | / | 1986 | 22 | γ | + | Resapath |
|  | 11535 | Mastitis | 2000 | 82 | γ | + | Resapath |
|  | 12538 | Mastitis | 2001 | 61 | γ | + | Resapath |
|  | 19623 | Mastitis | 2007 | 38 | γ | + | Resapath |
|  | 20576 | Mastitis | 2007 | 61 | α | + | Resapath |
|  | 20965 | Mastitis | 2008 | 29 | γ | + | Resapath |
|  | 19168 | Mastitis | 2007 | 1 | γ | + | Resapath |
|  | 20222 | Mastitis | 2007 | 74 | γ | + | Resapath |
|  | 16193 | Mastitis | 2007 | 42 | γ | + | Resapath |
|  | 19608 | Mastitis | 2007 | 1 | γ | + | Resapath |
| *S. dysgalactiae* subsp. *equisimilis* | 20591 | Synovial fluid's sampling equine | 2007 | 61 | β | + | Resapath |
| *S. bovis* | 7434 | Mastitis | 1993 | 82 | γ | + | Resapath |
|  | 1052 | Lung disease | 1985 | 49 | γ | - | Resapath |
|  | 1052 (pOri23-camp^515^) | / | / | / | γ | + | This work |
| *S. dysgalactiae* subsp. *dysgalactiae* | 24084 | / | 2010 | 50 | α | + | Resapath |
|  | 593 | / | 1984 | 22 | γ | - | Resapath |
|  | 593 (pOri23-camp^515^) | / | / | / | γ | + | This work |
| **Plasmids** | pOri23 | *ermAM ori* ColE1 *P23* | / | / | / | / | [52] |
|  | pOri23-camp^515^ | *ermAM* ori ColE1 *P23* SAL_2074 | / | / | / | / | This work |
|  | pG+host9 | pWV01-type thermosensitive replication origin from pVE6002, Erm^r^ | / | / | / | / | [47] |
|  | pG+host9spc | plasmid derived from pG+host9, Spc^r^ | / | / | / | / | X. Bellanger, pers. comm. |
