# Supplementary material for: Characterization of a New CAMP Factor Carried by an Integrative and Conjugative Element in Streptococcus agalactiae and Spreading in Streptococci
Source: PLoS One. 2012 Nov 9;7(11):e48918. doi: 10.1371/journal.pone.0048918 (PMC3494709; doi:10.1371/journal.pone.0048918)
Supplement: Table S2 — Primers used in this work. Restriction sites appear in bold. (DOC) [file pone.0048918.s005.doc]

**Supporting Information Table 2. Primers used in this work.** Restriction sites appear in bold.

| **Target gene** | **5’-3’ sequence** |
| --- | --- |
| SAG2026-1-***Hind*III** | AAAAA**AAGCTT**CATTCCATTTAATAACCATC |
| SAG2026-2-***Avr*II** | AAAAA**CCTAGG**TCACTCGTTGTTCTACAGTA |
| SAG2026-3-***Avr*II** | AAAAA**CCTAGG**AAATAGTGAATATCCCCA |
| SAG2026-4-***Eco*RI** | AAAAA**GAATTC** TGTTAGGATTGGAGTTTAG |
| CAMP factor ICE_*515_tRNA^Lys^* RT-PCR fwd | ATCATGTCGTAGTTAGTCAAGTTA |
| CAMP factor ICE_*515_tRNA^Lys^* RT-PCR Rev | TCCGAATCCCATGTCAAC |
| CAMP factor ICE_*515_tRNA^Lys^* ***Bam*HI** fwd | TTTTT**GGATCC**AAGTTATTTTGATTTAGGAGGG |
| CAMP factor ICE_*515_tRNA^Lys^* ***Nsi*I** Rev | TTTTT**ATGCAT**GAATATCGCTGTTGCCTT |
| Uberis factor U34322.1 fwd | AAGAGGAATGCTTATGGAA |
| Uberis factor U34322.1 rev | TCCACCAAATCTCTTCAAC |
| ICE_*515_tRNA^Ly^*^s^ integrase fwd | AAGCGTGAAGCTATGAATGA |
| ICE_*515_tRNA^Lys^* integrase rev | CTACCAGCTATGACAACTCCA |
| ICE_*SUB19608_tRNA^Lys^* integrase fwd | AAACGTGAAGCTATGAATGAAG |
| ICE_*SUB19608_tRNA^Lys^* integrase Rev | TTACCAGCTATCACAACACC |
| tRNA^Lys^ AAA Rev | GGTTATTGCTGTGGCATACG |
| HMPREF9319_0116 fwd | CCCTGCAAGCAAATGAGG |
| HMPREF9964_2030 fwd | CCTCACTTTGCCATTCCT |
| CAMP factor ICE_*515_tRNA^Lys^* Rev | TCCGAATCCCATGTCAAC |
